# Protocol for a scoping review of sepsis epidemiology

**DOI:** 10.1186/s13643-022-02002-6

**Published:** 2022-06-19

**Authors:** M. Elizabeth Wilcox, Marietou Daou, Joanna C. Dionne, Peter Dodek, Marina Englesakis, Allan Garland, Claire Lauzon, Osama Loubani, Bram Rochwerg, Manu Shankar-Hari, Kednapa Thavorn, Andrea C. Tricco

**Affiliations:** 1 Department of Medicine, University Health Network, Toronto, ON Canada; 2 Interdepartmental Division of Critical Care Medicine, University of Toronto, Toronto, ON Canada; 3 Department of Medicine, McMaster University, Hamilton, ON Canada; 4 Department of Health Research Methods, Evidence and Impact, McMaster University, Hamilton, ON Canada; 5 Department of Medicine, University of British Columbia, Vancouver, BC Canada; 6 Center for Health Evaluation and Outcome Sciences, St. Paul’s Hospital and University of British Columbia, Vancouver, BC Canada; 7 Library & Information Services, University Health Network, Toronto, ON Canada; 8 Professor of Medicine, and Community Health Sciences, University of Manitoba, Winnipeg, MB Canada; 9 Departments of Emergency Medicine and Critical Care, Dalhousie University, Halifax, NS Canada; 10 Chair of Translational Critical Care Medicine, Centre for Inflammation Research, The University of Edinburgh, Edinburgh, UK; 11 School of Epidemiology and Public Health, University of Ottawa, Ottawa, ON Canada; 12 Ottawa Hospital Research Institute, The Ottawa Hospital, Ottawa, ON Canada; 13 Li Ka Shing Knowledge Institute, St. Michael’s Hospital, Unity Health Toronto, Toronto, ON Canada; 14 Epidemiology Division and Institute of Health Policy, Management, and Evaluation, Dalla Lana School of Public Health, University of Toronto, Toronto, ON Canada; 15 Queen’s Collaboration for Health Care Quality Joanna Briggs Institute Centre of Excellence, Queen’s University, Kingston, Canada

**Keywords:** Sepsis, Scoping review, Organ failure, Infection, Systemic inflammatory response syndrome, Sequential Organ Failure Assessment

## Abstract

**Introduction:**

Sepsis is a common, life-threatening syndrome of physiologic, pathologic, and biochemical abnormalities that are caused by infection and propagated by a dysregulated immune response. In 2017, the estimated annual incidence of sepsis around the world was 508 cases per 100,000 (95% confidence interval [CI], 422–612 cases per 100,000), however, reported incidence rates vary significantly by country. A scoping review will identify knowledge gaps by systematically investigating the incidence of sepsis.

**Methods and analysis:**

This scoping review will be guided by the updated JBI (formerly Joanna Briggs Institute) methodology. We will search the following electronic databases: MEDLINE, EMBASE, CINAHL, and Cochrane Database of Systematic Reviews/Central Register of Controlled Trials. In addition, we will search websites of trial and study registries. We will review titles and abstracts of potentially eligible studies and then full-texts by two independent reviewers. We will include any study that is focused on the incidence of sepsis or septic shock in any population. Data will be abstracted independently using pre-piloted data extraction forms, and we will present results according to the Preferred Reporting Items for Systematic Reviews and Meta-analysis Protocols Extension for Scoping Reviews.

**Ethics and dissemination:**

The results of this review will be used to create a publicly available indexed and searchable electronic registry of existing sepsis research relating to incidence in neonates, children, and adults. With input from stakeholders, we will identify the implications of study findings for policy, practice, and research. Ethics approval was not required given this study reports on existing literature.

**Supplementary Information:**

The online version contains supplementary material available at 10.1186/s13643-022-02002-6.

## Strengths and limitations

- By cataloging what is known about the epidemiology of sepsis, we will clarify gaps in our understanding of its incidence and distribution.
- By including patients of all ages, this review may uncover differences in the epidemiology of sepsis between neonatal, pediatric, and adult populations.
- By describing rates across the last three decades, we may better understand if evolving definitions of sepsis have influenced reported incidence rates.
- Through the inclusion of all languages, we may better delineate differences in the international reporting of incidence rates of sepsis.

## Introduction

Though consensus-based definitions of sepsis have evolved over the past 30 years [1–3], the core element has remained constant; the most recent, SEPSIS-3 definition, describes it as “life-threatening organ dysfunction caused by a dysregulated host response to infection” [3]. Despite efforts at improving its clinical definition, physicians struggle to identify sepsis [4]. For example, among 2579 critically ill patients with presumed sepsis, in post hoc assessment, 43% were judged unlikely to have had an infection [5]. Challenges in the clinical identification of sepsis threaten the accuracy of reported sepsis estimates.

The World Health Organization (WHO) estimates that sepsis affects over 30 million people each year, and is responsible for over 6 million deaths worldwide, including one million newborns [6]. Recently, for the first time, the Global Burden of Disease (GBD) Study included sepsis estimates in its reporting of cause of death data from over 100 million individual death records across 195 countries [7]. For 2017, it estimated 48.9 million incident cases of sepsis worldwide (implicit and explicit sepsis cases) and 11 million sepsis-related deaths. This would represent 19.7% of all global deaths [7]. Declining incidence was seen in nearly every location, with the highest age-standardized incidence of sepsis occurring in areas with the lowest sociodemographic index. Concerningly, however, there seems to be a general lack of national-level reporting of sepsis incidence, and in some cases, crude estimates based on applied modeling from all-cause mortality estimates are the only data available [7]. Furthermore, existing studies are heavily represented by high-income countries as compared to low- and middle-income countries where data may be less readily available. Such economic disparities may influence the reporting of vaccine preventable “infections” likely to impact sepsis incidence over time.

Vulnerable populations such as those who live in poverty, or who have less education, higher unemployment, visible minority status, or more functional limitations have higher mortality rates from sepsis [8]. Although these vulnerabilities may in part be explained by geographical barriers to preventative care and early diagnosis and treatment, further investigation is needed to understand the role of socio-demographic inequities and characteristics of at-risk populations. Furthermore, the study of both national and international differences in equity of access may reveal how socio-political conditions predispose certain individuals to the development of sepsis or influence sepsis-associated mortality. Accurate national data on sepsis may be used to establish healthcare policy and allocate healthcare resources.

The main objectives of this scoping review are (1) to describe the epidemiology of sepsis (i.e., incidence rate), (2) to report incidence rates over time, and (3) to identify existing knowledge gaps to inform and guide future research. This scoping review will catalog existing knowledge and identify gaps, and as such it will be a fundamental infrastructure in informing and guiding future research as well as policy work in sepsis.

## Methods and analysis

### Objectives

This is a protocol for a scoping review aimed at understanding what is known about the epidemiology of sepsis. Methods for inclusion and analysis of articles will be performed according to the updated JBI (formerly Joanna Briggs Institute) guide to scoping review methodology [9]. The main items in the Preferred Reporting Items for Systematic Reviews and Meta-analysis Protocols Extension for Scoping Reviews (PRISMA-ScR) [10, 11] guided the reporting of this protocol, and furthermore, it will be registered with Open Science Framework (https://osf.io/) upon acceptance.

The specific objectives of this scoping review are:

1. Identify the types of available evidence of the epidemiology of sepsis;
2. Clarify key concepts/definitions in the literature;
3. Examine how research is conducted in the field of sepsis epidemiology;
4. Identify and analyze knowledge gaps as fundamental infrastructure to inform and guide future research, policy work, ultimately with the aim of facilitating future sepsis research; and
5. Inform the conduct of a systematic review examining the epidemiology of sepsis.

### Eligibility criteria

We will include citations if: (1) the study design is a non-randomized controlled trial, cohort study, case–control study, or cross-sectional study; (2) the study includes a population or subgroup of patients with defined sepsis or septic shock (e.g., regional or national); and (3) the population being described the report on the population incidence of sepsis (i.e., incidence). A preliminary exploratory review of studies examining sepsis will help refine the scope of the present protocol. We will not apply any publication date or language restrictions. Because our primary objective is to understand how the development of sepsis may vary among age groups, country of residence, socio-economic status, etc., there will be no eligibility criteria restricting the scoping review to specific populations.

### Literature search

In collaboration with an experienced health science librarian, we will search the following databases from inception: MEDLINE, EMBASE, CINAHL, and Cochrane Library [12]. To ensure reproducibility, each search strategy will be validated by a second medical librarian using the Peer Review of Electronic Search Strategies (PRESS) checklist [13, 14]. The proposed MEDLINE and EMBASE search strategies are outlined in the online supplementary appendix. All reference management will be performed in Covidence (Version © 2022, Melbourne, Australia). Reference lists of included studies and relevant reviews will be searched to identify additional relevant sources. Authors of primary sources will be contacted should any further information be required.

The unpublished literature (i.e., difficult to locate unpublished material) will also be searched [15]. Specifically, we will search Google and websites of agencies that fund, report or conduct sepsis studies, such as the World Health Organization. Web pages pertaining to the Center of Disease Control (CDC) and other sepsis organizations were also examined for the purpose of this review.

### Selection of sources/screening (level 1)

After removal of duplicates, titles and abstracts of all search results will be assessed for inclusion against eligibility criteria. Titles/abstracts will be identified as “include,” “exclude,” or “maybe.” We will screen in two stages: first titles and abstracts, then full texts, and screening at both stages will be completed independently and in duplicate by two reviewers from the screening team. Prior to the screening of titles and abstracts (level 1 screening), the citation screening form will be calibrated through pilot testing with a random sample of 50 citations from the literature search by two reviewers, independently. The reviewer team will then independently review titles and abstracts, meet to discuss discrepancies, and make modifications to the eligibility criteria and screening form, and true screening will commence when at least 75% agreement is reached [14]. Full texts will be reviewed for all studies marked as “include” by one or more reviewers and will be independently identified as either “include” or “exclude” by both reviewers. Discrepancies will be resolved by discussion and consensus between the two reviewers, or by a third-party decision.

### Data charting/extraction (level 2)

During this stage, we will collect key information about the selected articles using a predefined Data Extraction Form. The extraction form was first pilot-tested using five studies by two independent reviewers; the extracted data will include the following fields (Table 1). The research team will meet to ensure that all appropriate information is to be collected, at which time the form may be modified before true data charting commences. The two members of the team will be responsible for independently charting the data from each included article. To ensure inter-rater reliability, a 20% sample of included articles independently reviewed will then be compared by the two members of the research team. Discrepancies in extracted data will be discussed between reviewers until consensus is reached or by arbitration of a third reviewer, as required.

**Table 1:** Data charting form

Author and date	
Title of study	
Publication	
Country/region	
Study setting	
Study population (adult, pediatrics, neonatal, maternal or mixed)	
Study design	
Data source(s)	Database used for case numbers (e.g., prospective registry)
Definition of sepsis used or specifics with regards to case identification	Clinical definition used to identify cases of sepsis or administrative database codes (or combinations of codes) to identify cases of sepsis
Number of cases (sepsis/severe sepsis)	
Calendar year	
Study duration	
Sepsis incidence/100,000 persons	
Sepsis incidence/100,000 person years	
Severe sepsis incidence/100,000 persons	
Severe sepsis incidence/100,000 person years	
Septic shock incidence/100,000 persons^a^	
Septic shock incidence/100,000 person years^a^	
Comment(s)	

^a^For manuscripts published before the SEPSIS-3 definition

### Knowledge user engagement activities

We define knowledge user (KU) engagement as individuals who may be affected by the research findings. We have recruited scientists from the Canadian Sepsis Network, a network of Canadian researchers with areas of expertise that include basic science, health services research, and health policy and specifically, individuals with expertise in neonatology and pediatrics.

Partners will be engaged in the scoping review process including the refinement of study inclusion criteria as well as interpretation of findings to inform dissemination activities. Activities will be sequential, and findings will inform the next steps.

### Patient and public involvement

No patient was involved.

### Synthesis

Scoping reviews do not include quantitative synthesis of results, but rather characterize the existing literature and identify research gaps. A narrative report will be produced to summarize the extracted data around the following outcomes for each of the populations studied (neonates/maternal, pediatrics, and adults): region of study, data sets used, incidence, associated definitions used, and changes reported in incidence over time. These results will be described in relation to the research question and in the context of the overall study purpose. Descriptive frequencies of the aims/results of the findings will be reported, and visually mapped to display the possible concentrations of sepsis research exploring incidence among certain populations and geographical regions. Gap identification will detect areas, such as countries that lack data on the incidence of sepsis in specific populations (e.g., neonates), and if there is a paucity of data on significant sepsis-related conditions.

## Ethics and dissemination

Since the scoping review will synthesize information from available publications, it does not require ethical approval. The end-users identified, in addition to individual sepsis investigators, include the Canadian Sepsis Foundation, British Columbia Patient Safety and Quality Council, Canadian Critical Care Society, Canadian Critical Care Trials Group, Global Sepsis Alliance, Chief Medical Officers of Health in Canadian provinces, Canadian Association of Emergency Physicians, Canadian Critical Care Translational Biology Group and the Network of Canadian Researchers.

Potential barriers to study uptake include (1) lack of awareness and (2) lack of time. To circumvent these barriers, an article reporting the results of the scoping review will be submitted for publication to a scientific peer-reviewed journal; the research team will conduct webinars reporting study findings, investigators will present results at national and international meetings, and lastly, an organized twitter campaign will further awareness of study results. In recognizing the barrier of a lack of time, a one-page summary tailored to different audiences will be crafted and made available. We will organize collated information into a publicly available indexed and searchable electronic registry of existing sepsis research. This registry will be publicly available through the Sepsis Research Network’s website (https://www.sepsiscanada.ca/).

## Supplementary Information

**Additional file 1: Appendix 1.** Search strings for Medline and EMBASE.

## Data Availability

Not applicable.
