# Supplementary material for: Protocol for a scoping review of sepsis epidemiology
Source: Syst Rev. 2022 Jun 19;11:125. doi: 10.1186/s13643-022-02002-6 (PMC9208121; doi:10.1186/s13643-022-02002-6)
Supplement: Supplementary file 1 — Additional file 1: Appendix 1. Search strings for Medline and EMBASE. [file 13643_2022_2002_MOESM1_ESM.pdf]

Appendix 1. Search strings for Medline and EMBASE.

MedlineALL

#	Searches
1	exp Sepsis/
2	Shock, Septic/
3	exp Systemic Inflammatory Response Syndrome/
4	sepses.ti,ab,kw.
5	sepsis.ti,ab,kw.
6	septic.ti,ab,kw.
7	septicaemi*.ti,ab,kw.
8	septicemi*.ti,ab,kw.
9	(sirs and inflam*).ti,ab,kw.
10	systemic inflammatory response syndrome.ti,ab,kw.
11	or/1-10 [Sepsis]
12	Epidemiology/
13	Epidemiologic Factors/
14	Epidemiologic Measurements/
15	Epidemiologic Methods/
16	Epidemiologic Research Design/
17	Epidemiologic Studies/
18	Epidemiological Monitoring/
19	epidemiolog*.ti,ab,kw.
20	Sepsis/ep [Epidemiology]
21	Shock, Septic/ep [Epidemiology]
22	Systemic Inflammatory Response Syndrome/ep
23	or/12-22 [Epidemiology]
**24**	**11 and 23 [Sepsis + Epidemiology]**
25	Incidence/
26	(incidence or incidences).ti,ab,kw.
27	Incidence Study/
28	Prevalence/
29	Prevalence Study/
30	prevalence.ti,ab,kw.
31	prevalent.ti,ab,kw.
32	(frequency adj8 disease?).ti,ab,kw.
33	(distribution adj8 disease?).ti,ab,kw.

34	(pattern? adj8 disease?).ti,ab,kw.
35	"Global Burden of Disease"/ [New MeSH 2017]
36	Global Health/
37	(global and (burden? adj3 disease?)).ti,ab,kw.
38	global health.ti,ab,kw.
39	world health.ti,ab,kw.
40	or/25-39 [Distribution: Incidence or Prevalence or Frequency or Pattern]
**41**	**24 and 40 [Sepsis + Epidemiology + Distribution]**
	Limits applied:
42	limit 41 to (english or french)
43	42 not (animal or animals or ape or apes or baboon or baboons or bat or bats or bird or birds or boar or boars or bonobo or bonobos or bovine or camel or camels or canine or canines or cat or cats or cattle or chicken or chickens or chimpanzee or chimpanzees or dog or dogs or dromedary or dromedaries or duck or ducks or equine or equines or feline or felines or ferret or ferrets or frog or frogs or fowl or fowls or goat or goats or hare or hares or hen or hens or horse or horses or lamb or lambs or livestock or macaque or macaques or mandrill or mandrills or mice or mink or minks or monkey or monkeys or mouse or murine or ovine or pig or pigs or piglet or piglets or poultry or porcine or orangutan or orangutans or rabbit or rabbits or rat or rats or rodent or rodents or sheep or swine or tamarin or tamarins or tiger or tigers or veterinary or veterinarian or veterinarians or waterfowl or waterfowls or weasel or weasels or veterinar* or fish or shellfish).ti,jw.
44	limit 42 to "humans only (removes records about animals)"
45	43 or 44 [Limiting to Human]

Embase

#	Searches
1	exp Sepsis/
2	Shock, Septic/
3	exp septic shock/ [Embase]
4	exp Systemic Inflammatory Response Syndrome/
5	sepses.ti,ab,kw.
6	sepsis.ti,ab,kw.
7	septic.ti,ab,kw.
8	septicaemi*.ti,ab,kw.
9	septicemi*.ti,ab,kw.
10	(sirs and inflam*).ti,ab,kw.
11	systemic inflammatory response syndrome.ti,ab,kw.
12	or/1-11 [Sepsis]
13	Epidemiology/
14	Epidemiologic Factors/
15	Epidemiologic Measurements/
16	Epidemiologic Methods/
17	Epidemiologic Research Design/
18	Epidemiologic Studies/
19	Epidemiological Monitoring/
20	epidemiolog*.ti,ab,kw.
21	Sepsis/ep [Epidemiology]
22	Shock, Septic/ep [Epidemiology]
23	exp septic shock/ep [Embase]
24	exp Systemic Inflammatory Response Syndrome/ep
25	or/13-24 [Epidemiology]
**26**	**12 and 25 [Sepsis + Epidemiology]**
27	Incidence/
28	(incidence or incidences).ti,ab,kw.
29	(Incidence adj2 (study or studies)).ti,ab,kw.
30	Prevalence/
31	Prevalence Study/
32	prevalence.ti,ab,kw.
33	prevalent.ti,ab,kw.
34	(frequency adj8 disease?).ti,ab,kw.
35	(distribution adj8 disease?).ti,ab,kw.
36	(pattern? adj8 disease?).ti,ab,kw.
37	"Global Burden of Disease"/ [New MeSH 2017]
38	global disease burden/ [Embase]
39	Global Health/

40	(global and (burden? adj3 disease?)).ti,ab,kw.
41	global health.ti,ab,kw.
42	world health.ti,ab,kw.
43	or/27-42 [Distribution: Incidence or Prevalence or Frequency or Pattern]
**44**	**26 and 43 [Sepsis + Epidemiology + Distribution]**
	Limits applied:
45	limit 44 to (english or french)
46	45 not (animal or animals or ape or apes or baboon or baboons or bat or bats or bird or birds or boar or boars or bonobo or bonobos or bovine or camel or camels or canine or canines or cat or cats or cattle or chicken or chickens or chimpanzee or chimpanzees or dog or dogs or dromedary or dromedaries or duck or ducks or equine or equines or feline or felines or ferret or ferrets or frog or frogs or fowl or fowls or goat or goats or hare or hares or hen or hens or horse or horses or lamb or lambs or livestock or macaque or macaques or mandrill or mandrills or mice or mink or minks or monkey or monkeys or mouse or murine or ovine or pig or pigs or piglet or piglets or poultry or porcine or orangutan or orangutans or rabbit or rabbits or rat or rats or rodent or rodents or sheep or swine or tamarin or tamarins or tiger or tigers or veterinary or veterinarian or veterinarians or waterfowl or waterfowls or weasel or weasels or veterinar* or fish or shellfish).ti,jw.
47	limit 45 to "humans only (removes records about animals)"
48	46 or 47 [Limiting to Human]
49	limit 48 to (conference abstracts or (books or chapter or conference abstract or "conference review") or (book or book series or conference proceeding))
50	48 not 49
